# Study on Tim3 Regulation of Multiple Myeloma Cell Proliferation *via* NF-κB Signal Pathways

**DOI:** 10.3389/fonc.2020.584530

**Published:** 2020-11-19

**Authors:** Zhaoyun Liu, Chenhuan Xiang, Mei Han, Nanhao Meng, Jingyi Luo, Rong Fu

**Affiliations:** Department of Hematology, Tianjin Medical University General Hospital, Tianjin, China

**Keywords:** apoptosis, proliferation, NF-κB, bortezomib, TIM3, multiple myeloma

## Abstract

**Objective:**

As an important negative regulatory factor of immunological cells, Tim3 plays a regulating role in tumor immune microenvironment. The purpose of this study was to investigate the expression of Tim3 on MM cells and its effect on the proliferation and apoptosis of MM cells, as well as its potential mechanism.

**Methods:**

In this study, the expression of Tim3 was detected on myeloma cells (CD38^+^CD138^+^ cells) of bone marrow by flow cytometry (FCM) from 167 patients with MM and 51 healthy donors as controls and making correlation analysis with related clinical indexes. *In vitro*, MM cell lines (RPMI-8226 and U266) were treated with Tim3 knock-down alone, bortezomib alone and combination of Tim3 knock-down and bortezomib, then cell proliferation, cell apoptosis and downstream signaling pathway were detected by CCK-8, FCM, RT-PCR and western blot.

**Results:**

The expression of Tim3 on myeloma cells in MM patients was significantly higher than normal control group and positively correlated with β_2_ microglobulin, creatine, and plasma cells of bone marrow, negatively correlated with hemoglobin and red blood cells. *In vitro*, we validated the high expression of Tim3 in RPMI-8226 and U266 cell lines. After Tim3 knock-down, the cell proliferation was inhibited and cell apoptosis was induced, the relative mRNA and protein expression of Tim3 and NF-κB signal pathway (PI3K, AKT, mTOR, NF-κB) were significantly decreased. Also, the cell proliferation was inhibited, cell apoptosis was increased, the relative mRNA and protein expression of NF-κB were decreased significantly in combination group than bortezomib or Tim3 knock-down group.

**Conclusions:**

The high expression of Tim3 on MM cells is associated with progression of MM patients. Tim3 maybe regulate the proliferation of MM cells *via* NF-κB signal pathway. Down-regulation of Tim3 expression can inhibit proliferation and induce apoptosis of MM cells, also has an additive inhibitory effect of bortezomib on NF-κB signaling pathway, then inhibit proliferation and induce apoptosis. Therefore, Tim3 may be a potential target for the treatment of MM.

## Introduction

Multiple myeloma (MM) is a malignant plasma cell disease characterized by the malignant proliferation of monoclonal plasma cells and the secretion of a large number of monoclonal immunoglobulin. It is one of the most common malignant tumors of blood system. The main clinical manifestations include anemia, recurrent infection, hypercalcemia, hyperviscosity syndrome, extensive bone destruction, and renal insufficiency. MM is an incurable malignant tumor and its incidence is rapidly increasing (1). At present, the therapeutic methods including proteasome inhibitors, immunosuppressants, monoclonal antibodies, histone deacetylase inhibitors, and autologous stem cell transplantation have shown remarkable curative effect. However, the prognosis of MM is still poor because of its high heterogeneity and drug resistance, so new treatment methods are still urgently needed (2, 3).

T-cell immunoglobulin and mucin domain-3(Tim3), as an important negative regulatory factor of cellular immunity, was first discovered on the surface of differentiated Th1 cells in 2002 (4), with common structural features including an N-terminal immunoglobulin (Ig)-like domain, a mucin domain, a transmembrane domain, and a cytoplasmic region with tyrosine phosphorylation motif(s). Tim3 is expressed selectively in T cells (Th1, Th17, CTL, Treg), dendritic cells (DCs), monocytes, mast cells, macrophages, NK cells, and other immune cells (5–10). Tim3 is expressed on Th1, Th17, CTL cells, and promotes the apoptosis of Th1, Th17, and the function exhaustion of CTL cell by binding to its ligand galectin-9, thus inhibiting antitumor immunity and leading to tumor immune escape (11). Tim3 is expressed on Treg cells, Tim3^+^Treg cells play an immunosuppressive role by secreting inhibitory cytokines IL-10 (8, 12). Tim3 is expressed on tumor-infiltrating DCs and inhibits innate immunity by binding to its another ligand high mobility family protein 1(HMGB1) competitively with nucleic acids, thus leading to tumor immune escape (13). Tim3 can inhibit immune response indirectly by inducing proliferation of myeloid-derived suppressor cells (MDSCs), which is related to the poor prognosis of tumors (14). In conclusion, Tim3 plays a crucial role in inhibiting the antitumor immune response (15).

Tim3 is expressed not only on immune cells but also in tumor cells. Among solid tumors, Tim3 is expressed on non-small cell lung cancer, esophageal squamous cell carcinoma, and its high expression on tumor-infiltrating lymphocytes (TILs) is associated with poor tumor prognosis (16, 17). Tim3 is highly expressed on cervical cancer cells and can be as an independent factor predicting the tumor prognosis, Tim3 down-regulation can inhibit the migration and invasion of cervical cancer cells (18). Tim3 is also highly expressed on prostate cancer cells and plays an important role in the development of prostate cancer, as a potential therapeutic target (19). Human hepatoma contains a high proportion of Tim-3 expressed hepatocytes, and that the overexpression of hepatocyte-speciﬁc Tim3 promotes tumor cell growth, whereas Tim3 inhibition on malignant hepatocytes by anti-Tim3 antibodies and in Tim-3 knockout mice through activating NF-κB phosphorylation by hepatocyte-Tim3 receptor mechanistically (20). Among blood system tumors, the overexpression of Tim3 on leukemic stem cells in acute myeloid leukemia (AML) can activate PI3K/AKT/mTOR and NF-κB signaling pathways (21). The overexpression of Tim3 on hematopoietic stem cells in myelodysplastic syndrome (MDS) is associated with malignant biological characteristics that make it abnormal differentiation and excessive proliferation, so Tim3 may be the marker for identifying malignant clonal cells or potential therapeutic targets for MDS (22). Tim3 is highly expressed on lymphocytes in MM, which may be associated with cellular immunodeficiency (23). But the studies on Tim3 in MM are rare, especially the expression and mechanism of Tim3 in MM cells have not been clearly described.

## Patients and Methods

### Patients

A total of 167 patients with MM and 51 healthy donors as controls were selected from the Department of Hematology of Tianjin Medical University General Hospital (Tianjin, China) between August 2017 and January 2019, and diagnosed according to the criteria for MM set out by the the International Myeloma Working Group (IMWG) (24), including 91 men and 76 women (median age: 59 years, range: 37–85 years), divided into two groups: group A including 88 newly diagnosed and clinical relapse patients (median age: 62 years, range: 37–82 years), group B including 79 patients with very good partial response (VGPR) and above after treatment (median age: 58 years, range: 46–85 years). The normal control group was group C, including 23 males and 28 females (median age: 56 years, range: 17–82 years) (show in **Table 1**). The research scheme has been approved by the Ethics Committee of Tianjin Medical University, and all patients have signed informed consent.

**Table 1:** Baseline characteristics of MM patients.

	Initial treatment/recurrence MM	Remission MM
	n(n/N)(%)	n(n/N)(%)
N	88	79
Sex		
Male	55(62.5)	36(45.6)
Female	33(37.5)	43(54.4)
Age		
<65 years	53(60.2)	57(72.2)
≥65 years	35(39.8)	22(27.8)
Median(range)	62(37-82)	58(46-85)
ISS stage		
I	12(13.6)	24(30.4)
II	32(36.4)	21(26.6)
III	44(50.0)	34(43.0)
M protein type		
IgG type	56(63.6)	31(39.2)
IgA type	18(20.5)	13(16.5)
IgM type	3(3.4)	2(2.5)
Light chain type	8(9.1)	17(21.5)
Non-secretary type	3(3.4)	16(20.3)

### Cell Culture 

Human MM cell lines (U266, RPMI-8226 and OPM2) were purchased from the Peking Union Cell Bank (Beijing, China). Which were cultured in RPMI 1640 medium (Solarbio, Beijing, China) containing 15% fetal calf serum (FBS, Gibco, California, USA) with 100µg/mL penicillin (Gibco) and 100 units/ml streptomycin (Gibco) in a humidifed incubator (37.5°C, 5% CO_2_).

## Flow Cytometry (FCM) 

The expression of Tim3 on myeloma cells or CD38^+^CD138^+^ (25, 26) cells were detected by FCM. Each table was divided into control tube and experimental tube. CD38-PE, CD138-FITC and IgG1-APC (BD Bioscience, USA) were added to the control tube, CD38-PE, CD138-FITC and Tim3-APC were added to the experimental tube, then 100ul samples were added to each tube, mixing and incubating for 15 min in dark, then 1ml hemolysin was added to each tube, mixing and incubating for 8 min in dark, centrifugating at 1,500 rpm for 5 min, discarding supernatant and 1ml PBS was added to each tube, mixing and centrifugating at 1,500 rpm for 5 min, also discarding supernatant. Finally, 300 ul PBS were added to each tube resuscitated cells waiting detection by Beckman CytoFLEX Flow Cytometer.

### Small Interfering RNA Transfection (SiRNA)

Cell lines were transfected with small interfering RNA (SiRNA) targeting the Tim3 (Gene Pharma, Shanghai, China) by the Lipofectamine ^TM^ 3000 Transfection Reagent (Invitrogen, Thermo Fisher Scientific, China) following the manufacturer’s instructions. 24 h before transfection, the cells were inoculated in Opti-MEM ^TM^ I Reduced Serum medium (Gibco, Thermo Fisher Scientific, China). After transfection for 6 h, the culture medium was replaced with PRMI1640, and 100 mM SiRNA was added followed by incubation for a further 24 h, 48 h, 72 h.

### Proliferation Assay (CCK8)

Cell viability was measured using the tetrazolium salt-based CCK-8 assay (Dojindo Molecular Technologies, Shanghai, China). The cell lines were randomly divided into four groups, negative control group (NC), Tim3 knock-down group, bortezomib treatment group, and combined group. The dosage of Tim3 SiRNA was fixed at 100nM, and he dosage of bortezomib was fixed at 10Nm. MM cell lines were seeded into 96-well plates at 1.0×10^5^ cell/mL in 100 µl complete medium. After 24 h, Tim3 SiRNA, bortezomib were added to. Plates were put at 37°C in 5% CO2, then 10 µl of CCK-8 reagent was added to the wells at 0 h, 24 h, 48 h, incubating for 2 h, the OD was read at 450 nm within 15 min. The experiment was repeated 3 times and each sample had 3 duplications. Cell viability was calculated using the following equation: cytotoxicity (%) = (OD450 of isogarcinol group/OD450 of control group) 100% (27).

### Cell Apoptosis Assays 

Cell apoptosis (28) was detected by flow cytometry and cells were stained with fluorescein-conjugated AnnexinV and PI (BD Bioscience, USA). After washing with phosphate-buffered saline (PBS) two times, cells were resuspended in binding buffer (10 mM N-2-hydroxyl piperazine-N0-2-ehane sulfonic acid/NaOH, pH 7.4, 140 mM NaCl, 2.5 mM CaCl_2_). One hundred microliters of cell suspension were incubated with 10 µl annexin V FITC and 10 µl PI for 20 min at room temperature in the dark. Cells were then detected by FCM within 30 min.

### Quantitative Real-Time Polymerase Chain Reaction (qRT-PCR) 

Total RNA was extracted from cells after acting for 48 h using the TRIzol reagent (Invitrogen, Carlsbad, CA, USA). One microgram of purifed total RNA was used for the real-time PCR analysis with the SuperScript First-Strand Synthesis System (Invitrogen). The qRT-PCR was performed in triplicates on the IQ5 (BIO-RAD, USA) with Super Real PreMix Plus SYBR Green (Tiangen Biotech, China) and primers (10 µM), and the amplified specific single product was validated by melt curve(primer sequence of genes and annealing temperature showed in **Table 2**). The results were calculated with the 2^-ΔΔCt^ method [(Ct, target gene Ct, β-actin) _sample_- (Ct target gene Ct, β-actin) _control_] after normalizing the data according to the β-actin mRNA expression (29).

**Table 2:** Primer sequence of gene and SiRNA.

Name	Sequence	Annealing temperature
Tim3	F 5’-CTGCTGCTGCTGCTGCTACTAC-3’ R 5’-TGAGCACCACGTTGCCACATTC-3’	58.3°C
PI3K	F 5’-ATCGCTCTGGCCTC ATTGAAGTTG-3’ R 5’-ATGGCTCGGTCCAGGTCATCC-3’	61.3°C
AKT	F 5’-GCAGGATGTGGACCAACGTGAG-3’ R 5’-GCAGGCAGCGGATGATGAAGG-3’	58.5°C
mTOR	F 5’-AACATCACCAACGCCACCACTG-3’ R 5’-GCCTCGCTCTCACTGTTGCTG-3’	59°C
NF-κB	F 5’-CAAGGACATGGTGGTCGGCTTC-3’ R 5’-CGCCTCTGTCATTCGTGCTTCC-3’	60.0°C
β-actin	F 5’-CCTGGCACCCAGCACAAT-3’ R 5’-GGGCCGGACTCGTCATAC-3’	–
Tim3-homo-451	F 5’-GUGCUCAGGACUGAUGAAATT-3’ R 5’-UUUCAUCAGUCCUGAGCACTT-3’	–
Tim3-homo-750	F 5’-GAGCCUCCCUGAUAUAAAUTT-3’ R 5’-AUUUAUAUCAGGGAGGCUCTT-3’	–
Tim3-home-311	F 5’-GGUCCUCAGAAGUGGAAUATT-3’ R 5’-UAUUCCACUUCUGAGGACCTT-3’	–
Negative control (NC)	F 5’-UUCUCCGAACGUGUCACGUTT-3’ R 5’-ACGUGACACGUUCGGAGAATT-3’	–
Negative control FAM	F 5’-UUCUCCGAACGUGUCACGUTT-3’ R 5’-ACGUGACACGUUCGGAGAATT-3’	–

### Western Blotting

Western blot evaluated the content of Tim3, PI3K, AKT, mTOR, NF-κB in cell for all groups extracts after 72 h of treatment. Cells were lysed with a lysis buffer (20 mM Tris-HCl, pH8.0,150 mM NaCl, 2 mM EDTA, 100 mM NaF, 1% NP40, 1 μg/ml leupeptin, 1 μg/ml anti-pain, and 1 mM phenylmethylsulfonyl fluoride), and the protein concentrations were determined with BCA protein assay kit (Dingguo Changsheng, Beijing, China). Proteins (30 μg) were separated using 8% SDS–poly-acrylamide gel.

Electrophoresis (SDS-PAGE). After electrophoresis, the proteins were transferred to a polyvinylidene diﬂuoride (PVDF) membrane (Solarbio, Beijing, China). PVDF membranes were blocked with a solution containing 5% skim milk and then incubated overnight at 4°C with the following antibodies: Tim3, PI3K, AKT, mTOR, NF-κB, GAPDH (Cell Signaling Technology, USA). After washing three times with Tris-buffered saline with Tween-20 (TBST, Solarbio, Beijing, China), each membrane was incubated for 1 h at room temperature with anti-rabbit IgG sheep antibody (Cell Signaling Technology, USA), also washing three times with TBST. Reactive proteins were visualized using a chemiluminescence kit (Solarbio, Beijing, China).

### Statistical Analysis

Statistical analyses were performed using IBM SPSS 19.0 statistical software. The results are expressed as means and S.D. Student’s t-test was used for the comparison between the two groups, and the one-way ANOVA analysis was used for the comparison between multiple groups. Spearman method was used for correlation analysis. A value of P < 0.05 was considered statistically significant (*means *p*<0.05, **means *p*<0.01, ***means *p*<0.001).

## Results

### Expression of Tim3 in Bone Marrow of MM Patients and Normal Healthy People and Its Clinical Correlation Analysis

**Figure 1:**
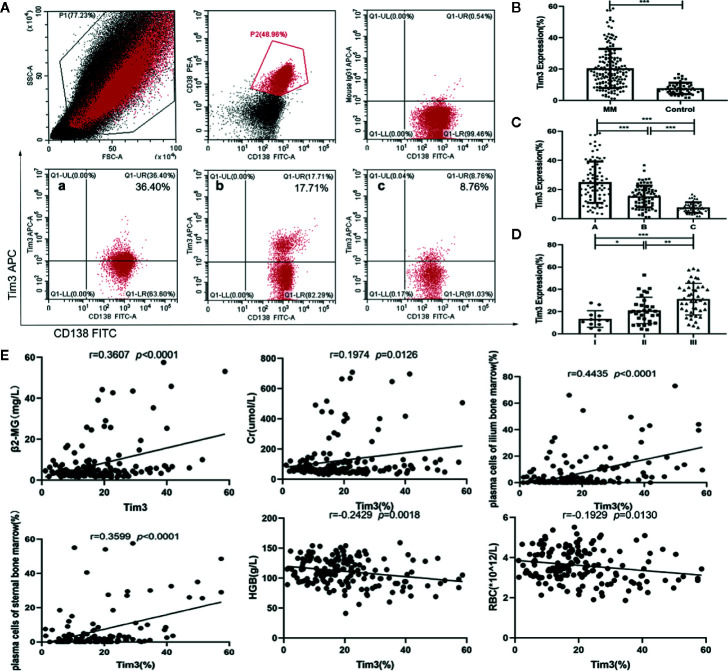
Expression of Tim3 in bone marrow of MM patients and normal healthy people and its clinical correlation analysis. **(A)** The expression of Tim3 on myeloma cells in MM patients(20.416±12.474%). (a) newly diagnosed patient, (b) patient with VGPR, (c) normal health control. **(B)** The expression of Tim3 in MM patients and nomal healthy control(7.665±3.592%). **(C)** The expression of Tim3 in initial treatment/recurrence MM, remission MM and control (25.047±14.185% vs. 15.257±7.454% vs. 7.665±3.592%). **(D)** The expression of Tim3 in ISS stage I, II, and III (13.338±7.505% vs. 21.023±11.850% vs. 31.167±14.242%). **(E)** Correlation analysis of Tim3 expression on myeloma cells and clinical indicators in MM patients. Shown are average values with standard deviations. * indicated p<0.05, ** indicated p<0.01, *** indicated p<0.001.

The expression of Tim3 on myeloma cells in MM patients was significantly higher than that on plasma cells of normal control group (20.416±12.474% vs. 7.665±3.592%, *p*<0.001: show in **Figures 1A, B**). Among MM patients, the expression of group A was higher than group B (25.047±14.185% vs. 15.257±7.454%, *p*<0.001), and the expression of both groups was significantly higher than that of normal control group (group C), *p*<0.001, (show in **Figure 1C**). Among group A, the expression of ISS II stage was higher than that of I stage (21.023±11.850% vs. 13.338±7.505%, *p*=0.046), and the expression of III stage was higher than that of II stage (31.167±14.242% vs. 21.023±11.850%, *p*=0.003), the expression of III stage was higher than that of I stage (31.167±14.242%, 21.023±11.850%, *p*<0.001) (show in **Figure 1D**). The expression of Tim3 was positively correlated with β_2_ microglobulin (β_2_-MG), creatine (Cr) and plasma cells of bone marrow (r=0.3607, *p*<0.0001: r=0.1974, *p*=0.0126; r=0.4435, *p*<0.0001; r=0.3599, *p*<0.0001), and negatively correlated with hemoglobin (HGB) and red blood cells (RBC; r=0.2429, *p*=0.0018; r=0.1929, *p*=0.0130) (show in **Figure 1E**).

### The Expression of Tim3 in MM Cell Lines

**Figure 2:**
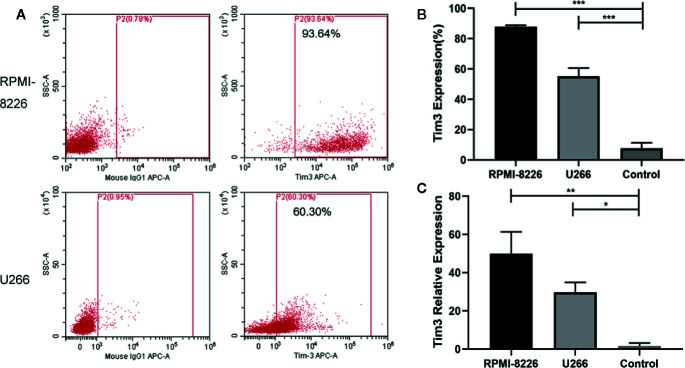
The expression of Tim3 in MM cell lines. **(A)** The expression of Tim3 in MM cell lines RPMI-8226 and U266. **(B)** The expression of Tim3 on RPMI-8226, U266, and control by flow cytometry (FCM). Control was from the plasma cells (CD38+ CD138+) of normal healthy people. **(C)** The expression of Tim3 in RPMI-8226, U266, and control by quantitative real-time polymerase chain reaction (qRT-PCR). Control was from normal healthy people. Shown are average values with standard deviations. Data are performed from 3 independent experiments. * indicated p<0.05, ** indicated p<0.01, *** indicated p<0.001.

The expression of Tim3 on MM cell lines RPMI-8226 and U266 was significantly higher than that in the normal control group (89.327±1.359% vs. 7.665±3.592%, *p*<0.001; 59.263±3.129% vs. 7.665±3.592%, *p*<0.001; show in **Figures 2A, B**). The relative mRNA expression of Tim3 in RPMI-8226 and U266 was significantly higher than that in normal control group (49.980±11.382 vs. 1.660 ±1.495, *p*=0.007; 29.673± 5.210 vs. 1.660 ±1.495, *p*=0.018; show in **Figure 2C**). Then cell lines were transfected with Tim3 SiRNA to knock down the expression of Tim3. The result showed that the knock-down effect of Tim3-homo-451 was the most obvious, and it could reach more than 50%, so Tim3-homo-451 was selected for subsequent transfection experiments.

### The Effect of Knocking Down Tim3 on the Apoptosis and Proliferation of MM Cell Lines 

**Figure 3:**
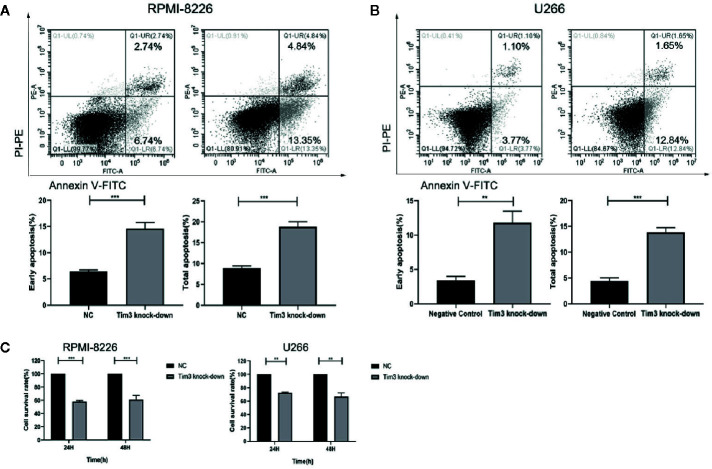
The effect of knocking down Tim3 on the apoptosis and proliferation of MM cell lines. **(A)** The effect of knocking down Tim3 on the apoptosis of RPMI-8226. **(B)** The effect of knocking down Tim3 on the apoptosis of U266. **(C)** The effect of knocking down Tim3 on the proliferation of RPMI-8226 and U266. Shown are average values with standard deviations. Data are performed from 3 independent experiments. ** indicated p<0.01, *** indicated p<0.001.

After Tim3 SiRNA transfection for 24 h, the early apoptosis rates and total apoptosis rates of RPMI-8226 were significantly higher than negative control (NC) group (14.623±1.130% vs. 6.420±0.291%, p<0.001; 18.823±1.211% vs. 8.930±0.477%, p<0.001) (show in **Figure 3A**). Similarly, the early apoptosis rates and total apoptosis rates of U266 were also higher than NC group (11.823±1.675% vs. 3.430±0.563%, p=0.001; 13.860±0.876% vs. 4.433±0.615%, p<0.001) (show in **Figure 3B**). The cell survival rates of MM cell lines decreased after knocking down Tim3, but without significant time dependence (p>0.05). The cell survival rates after transfection for 24 h and 48 h of RPMI-8226 were significantly lower than NC group (58.38±1.40% vs. 100.00±0.00%, p<0.001; 61.11±6.52% vs. 100.00±0.00%, p<0.001). The cell survival rates of U266 were significantly lower than NC group (72.58±1.07% vs. 100.00±0.00%, p=0.001; 66.88±5.43% vs. 100.00±0.00%, p=0.009) (show in **Figure 3C**).

### The Effects of Knocking Down Tim3 on Relative mRNA and Protein Expression of NF-κB Signal Pathway

**Figure 4:**
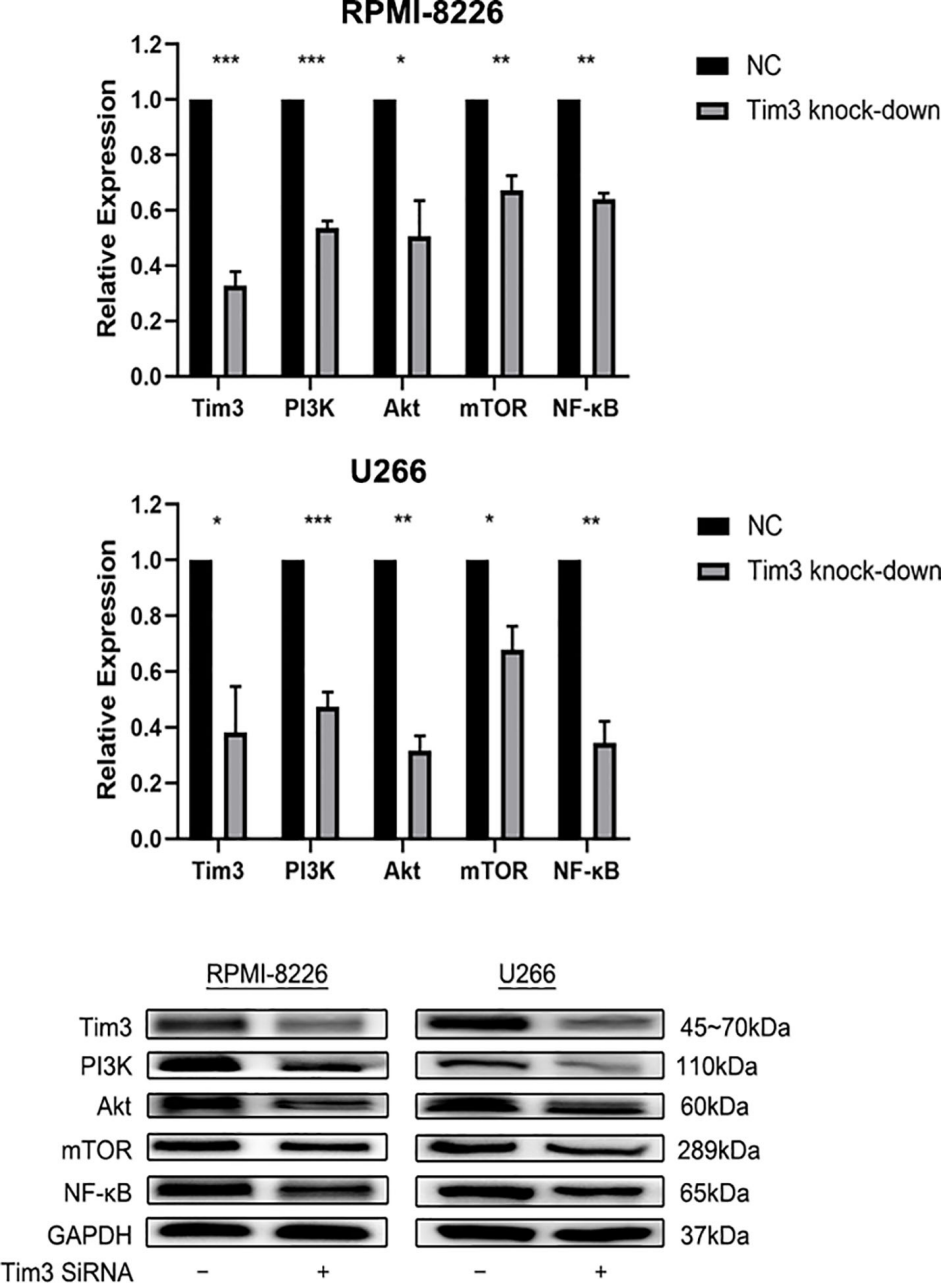
The effects of knocking down Tim3 on relative mRNA and protein expression of NF-κB signal pathway in RPMI-8226 and U266 cell lines. Shown are average values with standard deviations. Data are performed from 3 independent experiments.* indicated p<0,05, ** indicated p<0.01, *** indicated p<0.001.

The relative mRNA and protein expression of Tim3 and NF-κB signal pathway (PI3K, AKT, mTOR, NF-κB) were significantly decreased after knocking down Tim3 in RPMI-8226 and U266, p<0.05 (show in **Figure 4**).

### The Effects of Bortezomib Combined With Tim3 Knock-Down on the Apoptosis of MM Cell Lines 

**Figure 5:**
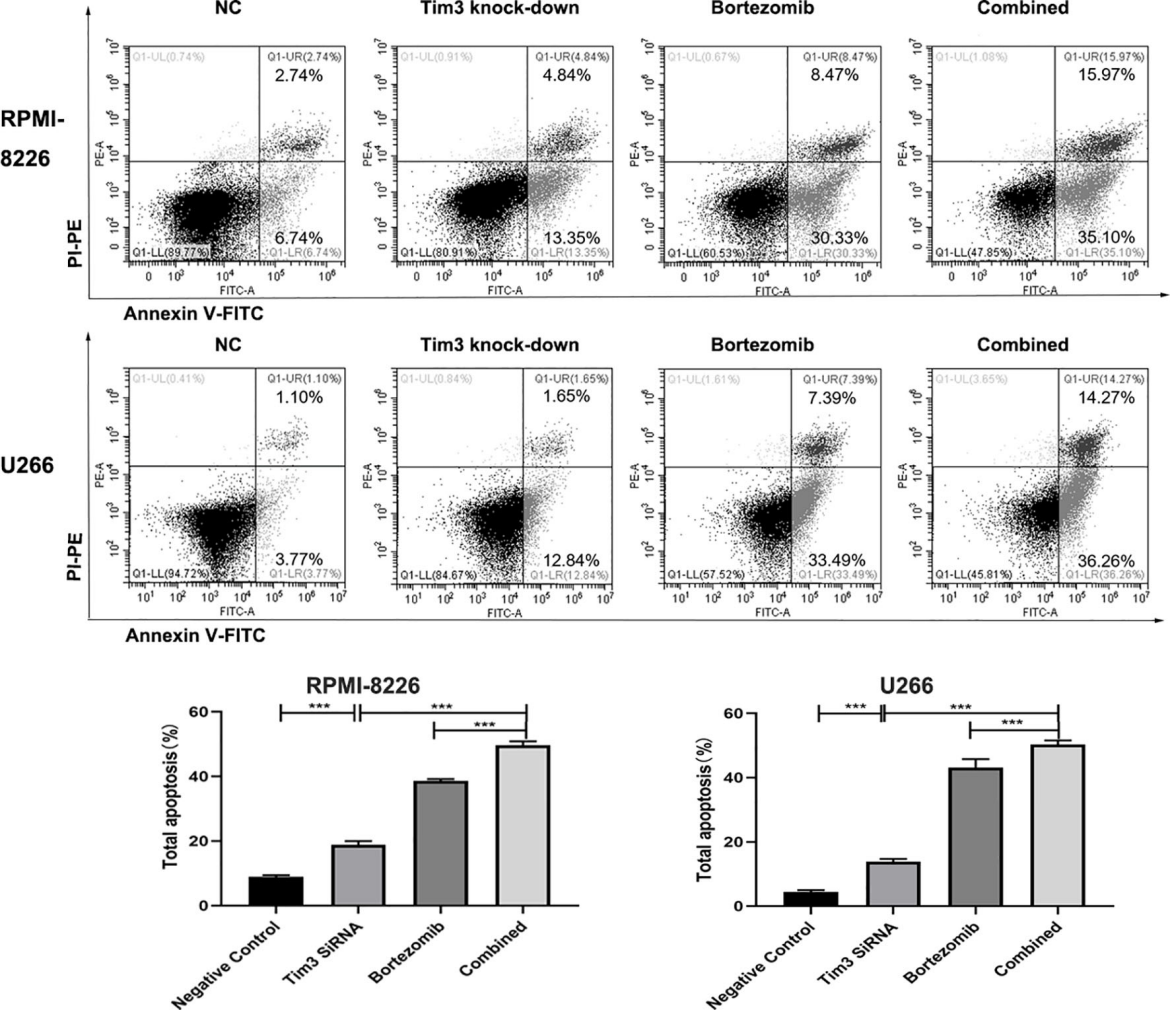
Cell apoptosis in the RPMI-8226 and U266 cell lines detected by flow cytometry using annexin V-Fluorescein isothiocyanate (FITC)/propidium iodide (PI) staining. Shown are average values with standard deviations. Data are performed from 3 independent experiments. *** indicated p<0.001.

The apoptosis rates of Tim3 knock-down group (18.223±1.211%), bortezomib group (38.610±0.56%) and combined group (49.683±1.201%) in RPMI-8226 were significantly higher than that of NC group (8.930±0.47%), the combined group was higher than Tim3 knock-down group and bortezomib group, p<0.001. Similarly, the apoptosis rates of Tim3 knock-down group (13.860±0.876%), bortezomib group (43.27±2.64%), and combined group (50.313±1.25%) were significantly higher than that of NC group (4.433±0.615%), the combined group was higher than Tim3 knock-down group and bortezomib group, p<0.001 (show in **Figure 5**).

### The Effects of Bortezomib Combined With Tim3 Knock-Down on the Proliferation of MM Cell Lines 

**Figure 6:**
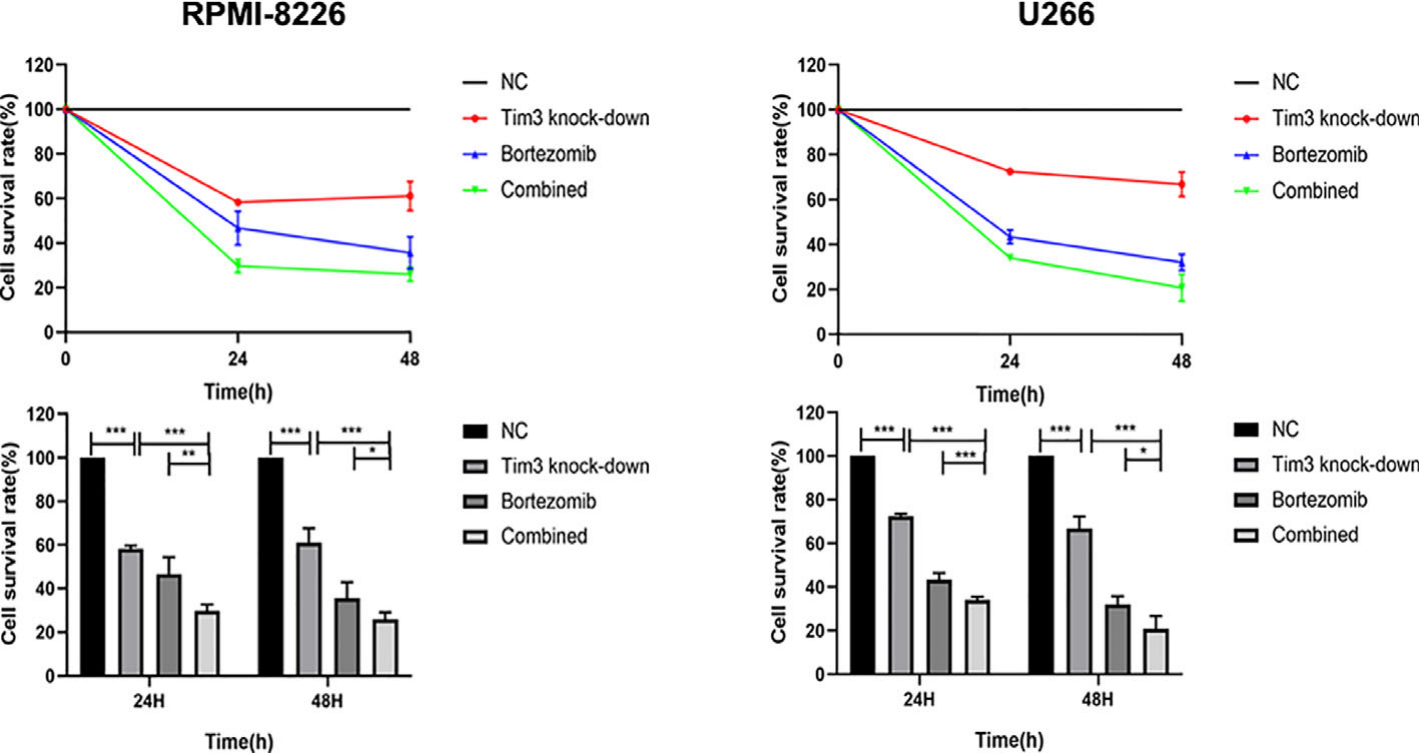
Cell proliferation in the RPMI-8226 and U266 cell lines. Shown are average values with standard deviations. Data are performed from 3 independent experiments. * indicated p<0.05, ** indicated p<0.01 and *** indicated p<0.001.

The cell survival rates of Tim3 knock-down group (58.38±1.40%), bortezomib group (46.78±7.66%) and combined group (29.74±2.87%) for 24 h in RPMI-8226 were significantly lower than that of NC group (100.00±0.00%), p<0.001, the combined group was lower than Tim3 knock-down group (p<0.001) and bortezomib group(p=0.001). The cell survival rates of Tim3 knock-down group (61.11±6.52%), bortezomib group (35.67±7.14%), and combined group (26.04±3.10%) for 48 h also decreased significantly, p<0.001, the combined group was lower than Tim3 knock-down group (p<0.001) and bortezomib group (p=0.049). The cell survival rates of Tim3 knock-down group (72.58±1.07%), bortezomib group (43.43±2.98%), and combined group (34.08±1.43%) for 24 h in U266 were significantly lower than that of NC group (100.00±0.00%), p<0.001, the combined group was lower than Tim3 knock-down group and bortezomib group, p<0.001. The cell survival rates of Tim3 knock-down group (66.88±5.43%), bortezomib group (32.10±3.64%), and combined group (20.75±5.86%) for 48h were significantly decreased, p<0.001, the combined group was lower than Tim3 knock-down group (p<0.001) and bortezomib group ( p=0.013) (show in **Figure 6**).

### The Effects of Bortezomib Combined With Tim3 Knock-Down on Relative mRNA and Protein Expression of NF-κB 

**Figure 7:**
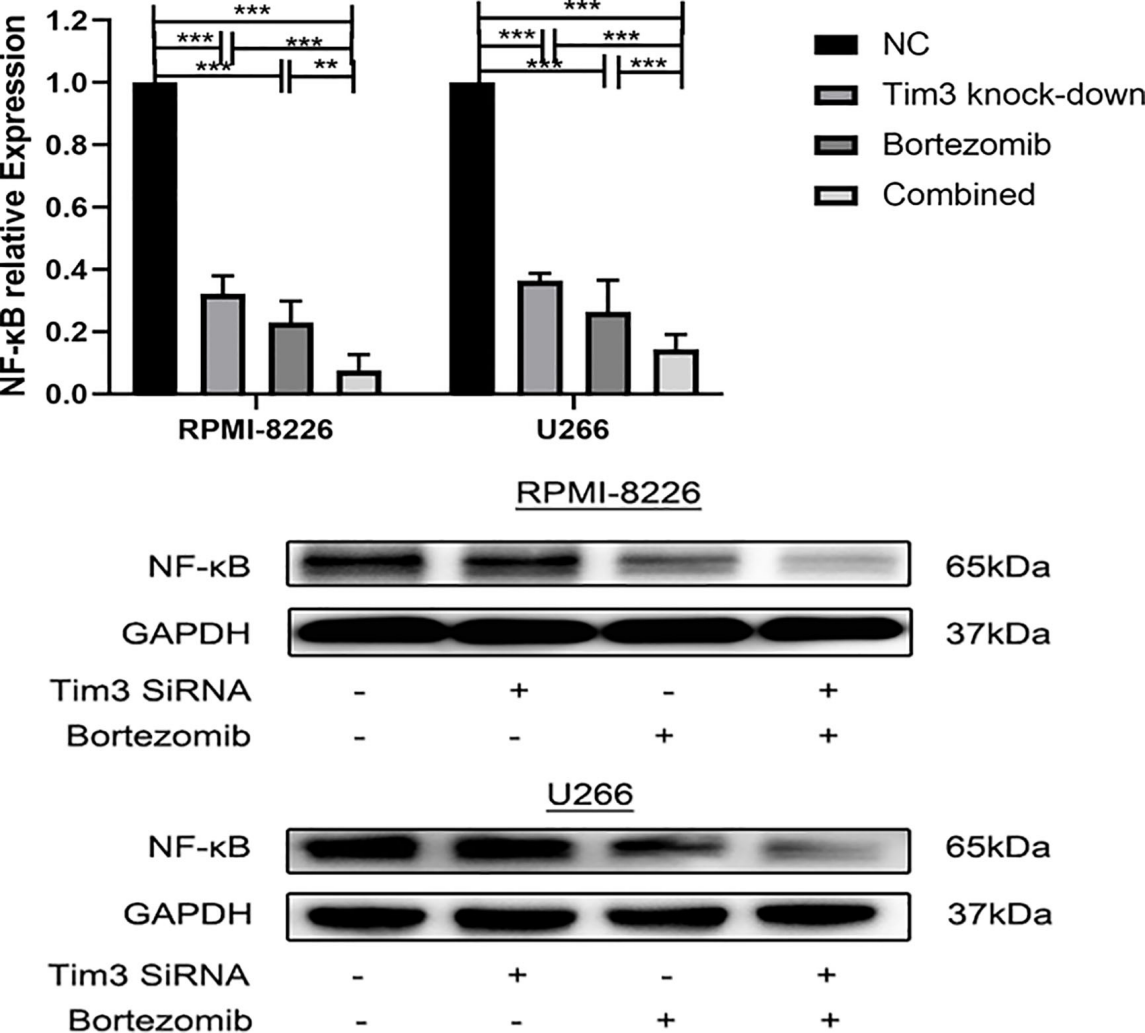
The effects of bortezomib combined with Tim3 knock-down on the relative mRNA and protein expression of NF-κB in RPMI-8226 and U266 cell lines. Shown are average values with standard deviations. Data are performed from 3 independent experiments.** indicated p<0.01, *** indicated p<0.001.

The relative NF-κB mRNA expression of Tim3 knock-down group (0.32±0.06), bortezomib group (0.23±0.07), and combined group (0.08±0.05) in RPMI-8226 was significantly decreased compared with that of NC group (1.00±0.00), p<0.001, the combined group was lower than Tim3 knock-down group (p<0.001) and bortezomib group (p=0.007). The relative NF-κB mRNA expression of Tim3 knock-down group (0.36±0.02), bortezomib group (0.24±0.01), and combined group (0.09±0.01) in U266 was significantly decreased compared with that of NC group (1.00±0.00), p<0.001, the combined group was lower than Tim3 knock-down group and bortezomib group, p<0.001. The NF-κB protein expression of Tim3 knock-down group, bortezomib group and combined group were significantly decreased compared with the NC group, and the combined group was lower than that of bortezomib group, p<0.05 (show in **Figure 7**).

## Discussion

Immunotherapy has gradually become a new method for tumor therapy. As a negative regulatory molecule, It has been confirmed that Tim3 can be expressed on a variety of immune cells, such as CD4^+^T cells (Th1, Th17), CD8^+^T cells, Treg cells, DCs, monocytes, mast cells, macrophages, NK cells, and plays an important role in tumor immunity in tumor microenvironment. Tim3 is expressed not only on immune cells, but also in tumor cells, such as melanoma, non-small cell lung cancer, esophageal cancer, hepatocellular carcinoma, cervical cancer, AML, etc, and is associated with tumor progression, poor prognosis. Down-regulation Tim3 can inhibit the proliferation, migration, and invasion and induce apoptosis of tumor cells (18).

MM is a malignant blood system tumor characterized by massive proliferation of monoclonal plasma cells in bone marrow. Malignant plasma cells directly infiltrate tissues and organs and their secreted M proteins lead to various clinical symptoms including anemia, bone pain, osteolytic bone destruction, hypercalcemia, and renal insufficiency (30). Studies have shown that Tim3 is overexpressed on T lymphocytes in MM patients and is involved in cellular immunodeficiency and tumor immune escape (23). While the expression and mechanism of Tim3 on MM cells have not been clearly elaborated. This study confirmed the high expression of Tim3 on MM cells, which is consistent with its overexpression on other tumor cells. We combined the newly diagnosed group and recurrence MM patients group because there is no significant difference in the patients clinical characteristics (**Supplementary Table 1**) and the expression of Tim-3 (**Supplementary Figure 1A**). Combining the two groups can increase the patients number we can analyse in our experiments,which will help us to explore Tim-3 in MM.As we separately analyzed the expression of tim-3 in the newly diagnosed and recurrence MM patients, we found significant difference in the expression of tim-3 in the newly diagnosed and recurrence MM patients group between stage I and III, stage II and III according to ISS stage (**Supplementary Figures 1B, C**).There is an obvious trend between stage I and II in both groups, which may due to the small number of specimens. Thus,those results support that the expression of tim-3 in plasma cell is different between MM and health control. The expression of Tim3 was positively correlated with β_2_-MG, Cr, and plasma cells of bone marrow and negatively correlated with HGB and RBC. Renal damage is the common and characteristic clinical manifestations in MM patients, Cr levels can reflect the extent of renal impairment in MM patients; β_2_-MG is a component of cell membrane protein, released to the blood circulation and excreted from the kidney when cells die. Elevated blood concentrations often indicate rapid tumor cell proliferation, disease progression. β_2_-MG is one of the important indicators of ISS stage, which can reflect tumor load and is associated with poor clinical prognosis (31). The number of plasma cells in bone marrow is one of the important indicators of diagnosis and prognosis in MM. Anemia is the most common clinical symptom in MM. The decrease of HGB can reflect the severity and prognosis of the disease (24). Meanwhile, the expression of Tim3 was positively correlated with ISS stage of MM patients. Thus, the expression of Tim3 on MM cells is related to the progression and poor prognosis of MM, which may be used as one of the indicators to MM clinical diagnosis and evaluate prognosis.

*In vitro*, the high expression of Tim3 on MM cell lines (RPNI-8226 and U266) was verified by both FCM and RT-PCR methods, which is consistent with the results of myeloma cells with MM patients. Then we knock down the expression of Tim3 in MM cell lines with Tim3 SiRNA to observe the effect of Tim3 on MM cells. The Tim3 SiRNA can be successfully transfected into MM cell lines by FCM verification. Tim3 SiRNA can down-regulate the expression of Tim3 gene and protein in the cell lines. The cell survival rates were detected by CCK-8, and it was found that Tim3 knock-down could inhibit the proliferation of MM cell lines (*p*<0.05), but without significant time dependence (*p*>0.05). The apoptosis is detected by FCM, it was found that Tim3 knock-down could induce apoptosis of MM cell lines, which increased both the early apoptosis rate and total apoptosis rate (*p*<0.05). It is suggested that Tim3 on MM cells can inhibit apoptosis and induce proliferation of MM cells.

The mechanism of Tim3 on MM cells is worth exploring. The importance of bone marrow microenvironment for growth and survival of MM cells is self-evident. MM cells interact with stromal and endothelial cells in bone marrow microenvironment to promote the production of related cytokines, which in turn can activate signaling pathways that promote the survival of MM cell, such as PI3K/AKT/mTOR signaling pathways, which are essential for growth, survival and resistance of MM cells (32, 33). NF-κB signaling pathway also plays an important role in the pathogenesis and treatment of MM (34). We predicted that the effect of Tim3 on MM cells is related to these signaling pathways. We found that the relative mRNA and protein expression NF-κB signal pathway (PI3K, AKT, mTOR, NF-κB) decreased (*p*<0.05) with down-regulation Tim3 of MM cells, suggesting that Tim3 may be a potential therapeutic target.

Bortezomib has been widely used in clinical treatment and has achieved good results, as a kind of proteasome inhibitor, with significant antitumor activity in MM patients. Bortezomib plays an important role in proliferation of MM cells by inhibiting NF-κB signaling pathway (35). So, we chose bortezomib to explore its interaction with Tim3 furtherly. We found that compared with bortezomib alone group and Tim3 knock-down alone group, the combined group had higher apoptosis rate and lower cell survival rate, *p*<0.05, suggesting that down-regulation of Tim3 has an additive effect of bortezomib on MM cells. In terms of mechanisms, the expression of NF-κB in bortezomib group decreased significantly compared with that of negative control group, which confirmed the inhibitory effect of bortezomib on NF-κB, while the expression of NF-κB in combined group decreased significantly compared with that of bortezomib alone, indicating that Tim3 could regulated proliferation of MM cells through NF-κB signal pathway. Down-regulation Tim3 has an additive effect of bortezomib on the NF-κB signal pathway and then inhibit proliferation and induce apoptosis of MM cells, suggesting that Tim3 may be a potential therapeutic target for MM. But, this experiment was based on the vitro cell level, which needs to be further studied at the vivo level, and the action mechanism of Tim3 on MM still needs to be further studied and perfected.

In summary, Tim3 is highly expressed on MM cells and can regulate the proliferation of MM cells through the NF-κB signaling pathway. Down-regulation of Tim3 can inhibit the proliferation of MM cells and induce their apoptosis. Down-regulation of Tim3 has an additive effect of bortezomib on MM cells. Tim3 may be a potential therapeutic target for MM.

## Data Availability Statement

The original contributions presented in the study are included in the article/**Supplementary Material**. Further inquiries can be directed to the corresponding author.

## Ethics Statement

The studies involving human participants were reviewed and approved by the Ethics Committee of Tianjin Medical University. The patients/participants provided their written informed consent to participate in this study.

## Author Contributions

ZL and CX performed experiments. MH analyzed data. NM and JL contributed to materials and analysis tools. RF helped to design experiments. All authors contributed to the article and approved the submitted version.

## Funding

The present study was supported by grants from the National Natural Science Foundation of China (grant nos. 81570106, 81400088, 81400085 and 81900131), the Tianjin Education Commission Research Project (grant nos. 2018KJ045 and 2018KJ043), and the Tianjin Municipal Natural Science Foundation (grant nos. 16ZXMJSY00180 and 18JCQNJC80400).

## Conflict of Interest

The authors declare that the research was conducted in the absence of any commercial or financial relationships that could be construed as a potential conflict of interest.
