# Supplementary material for: Study on Tim3 Regulation of Multiple Myeloma Cell Proliferation via NF-κB Signal Pathways
Source: Front Oncol. 2020 Nov 19;10:584530. doi: 10.3389/fonc.2020.584530 (PMC7710973; doi:10.3389/fonc.2020.584530)
Supplement: Supplementary file 1 [file DataSheet_1.docx]

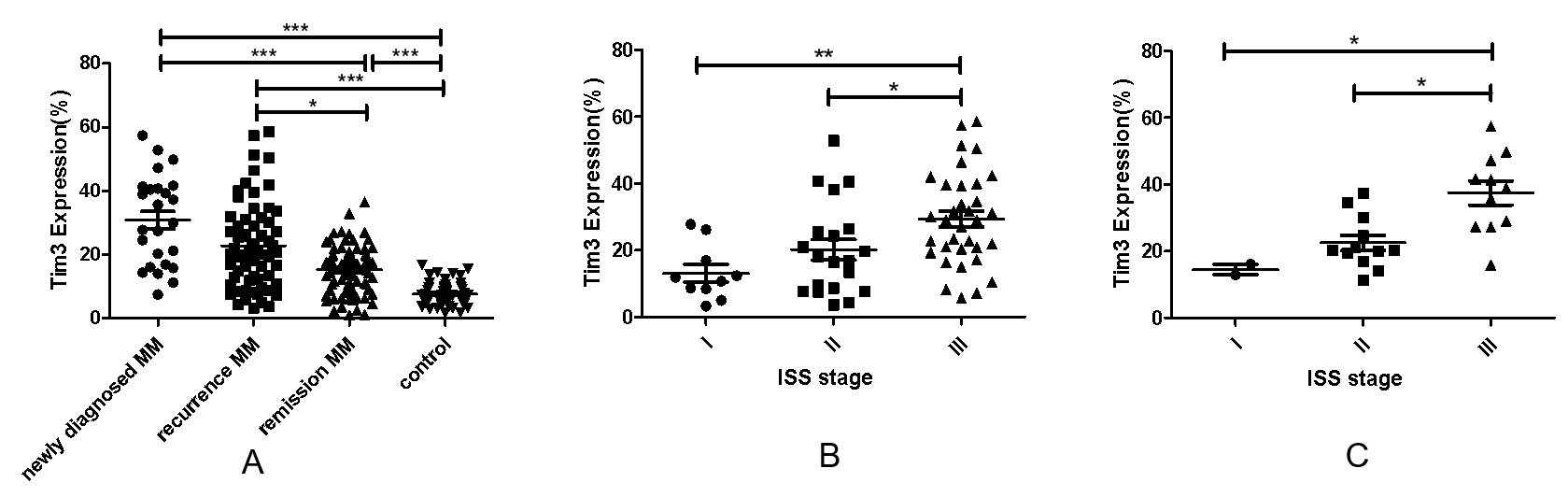


supplementary figure 1 A.Expression of Tim3 in bone marrow of newly diagnosed MM patients, recurrence MM, remission MM and normal healthy people. B.The expression of Tim3 on myeloma cells in recurrence MM patients according to ISS stage. C.The expression of Tim3 in newly diagnosed MM patients according to ISS stage. * indicated p<0.05, ** indicated p<0.01, *** indicated p<0.001.

|  | Initial treatment MM | recurrence MM | P value |
| --- | --- | --- | --- |
|  | n(n/N)(%) | n(n/N)(%) |  |
| N | 25 | 63 |  |
| Sex |  |  | 0.7658 |
| Male | 15(60) | 40(63.49) |  |
| Female | 10(40) | 23(36.51) |  |
| Age |  |  | 0.1802 |
| <65 years | 14(56) | 39(61.90) |  |
| ≥65 years | 11(44) | 24(38.10) |  |
| Median(range) | 59.5(37-65) | 62(44-82) |  |
| ISS stage |  |  | 1 |
| I | 2(8) | 10(15.87) |  |
| II | 12(48) | 20(31.75) |  |
| III | 11(44) | 33(52.38) |  |
| β2-Mg(mg/L) | 13.07±3.60 | 10.08±2.31 | 0.2235 |

supplementary table 1
